# Safety and effectiveness of ixekizumab in Japanese patients with psoriasis vulgaris, psoriatic arthritis, generalized pustular psoriasis, and erythrodermic psoriasis: Post‐marketing surveillance

**DOI:** 10.1111/1346-8138.17695

**Published:** 2025-03-13

**Authors:** Hideshi Torii, Akimichi Morita, Chie Yamamoto, Jiayi Dong, Mika Tsujimoto, Takashi Matsuo, Hitoe Torisu‐Itakura, Mamitaro Ohtsuki, Hidehisa Saeki

**Affiliations:** ^1^ Division of Dermatology Tokyo Yamate Medical Center Tokyo Japan; ^2^ Department of Geriatric and Environmental Dermatology Nagoya City University Graduate School of Medical Sciences Nagoya Japan; ^3^ Japan Drug Development and Medical Affairs, Eli Lilly Japan K.K. Kobe Japan; ^4^ Department of Dermatology Jichi Medical University Shimotsuke Japan; ^5^ Department of Dermatology Nippon Medical School Tokyo Japan

**Keywords:** ixekizumab, post‐marketing, psoriasis, safety, treatment effectiveness

## Abstract

We report findings from a post‐marketing study conducted from November 2016 to September 2022, which evaluated the safety and effectiveness of ixekizumab in Japanese patients with psoriasis under routine clinical practice for up to 52 weeks, and the incidence of serious infections and malignancies for up to 3 years. Of 804 patients in this analysis (67.9% male; median age, 54 years; mean disease duration, 11.8 years), 72.9%, 37.7%, 7.8%, and 3.7% had psoriasis vulgaris, psoriatic arthritis, pustular psoriasis, and erythrodermic psoriasis, respectively (subtypes not mutually exclusive). At 52 weeks, adverse events were reported in 203 patients (25.3%). Serious adverse events were reported in 36 patients (4.5%), including serious infections and infestations (*n* = 13, 1.6%). The incidence of serious infections and benign, malignant, and unspecified neoplasms was 0.8% (*n* = 5) and 0.6% (*n* = 4) respectively, at 3 years. Overall, 137 patients (17.0%) received Q2/Q2 treatment (160 mg starting dose, followed by 80 mg every 2 weeks from week 12); 550 patients (68.4%) received Q2/Q4 treatment (160 mg starting dose, followed by 80 mg every 2 weeks from weeks 2 to 12 and 80 mg every 4 weeks thereafter); and 117 patients (14.6%) discontinued before week 12 or received only one dose after week 12. A higher proportion of patients in the Q2/Q2 group had psoriatic arthritis (56.9% [*n* = 78]) compared with the Q2/Q4 group (32.9% [*n* = 181]). Among patients in the Q2/Q2 versus the Q2/Q4 dose groups, 21 (15.3%) and 141 (25.6%) respectively had adverse events and 2 (1.5%) and 32 (5.8%) respectively had serious adverse events. The mean Psoriasis Area and Severity Index score and body surface area percentage significantly decreased from baseline to week 52 for all psoriasis subtypes and by Q2/Q2 and Q2/Q4 ixekizumab doses (*p* < 0.01 or *p* < 0.001). Overall, the safety and effectiveness of ixekizumab in real‐world settings in Japan were similar to those reported in clinical trials.

## 1 INTRODUCTION

Psoriasis is a chronic, systemic inflammatory disease, primarily affecting the skin and joints.^1^ In Japan, psoriasis has a prevalence of 0.34% and is accompanied by psoriatic arthritis (PsA) in up to 23% of cases.^2^, ^3^, ^4^ The most common subtype is psoriasis vulgaris, characterized by dry, raised red patches of skin with silvery scales.^1^ Rarer clinical subtypes, such as generalized pustular psoriasis (GPP) and erythrodermic psoriasis (EP), are considered more severe forms of the disease and are potentially fatal if inadequately treated.^5^, ^6^ GPP is characterized by widespread sterile pustules, often accompanied by fever and systemic inflammation.^7^ EP is characterized by red, flaky skin over the majority of the body surface area (BSA), frequently accompanied by severe itching, pain, and multiple systemic symptoms.^8^ Psoriasis can be challenging to treat, as it is a chronic, relapsing disease, associated with significant comorbidities and a substantial disease burden.^9^, ^10^

Ixekizumab is a monoclonal antibody with high affinity and specificity to anti‐interleukin (IL)‐17A, a key pro‐inflammatory cytokine in the pathogenesis of psoriasis.^11^ Global phase 3 studies have demonstrated the efficacy and safety of ixekizumab in the treatment of moderate‐to‐severe plaque psoriasis for up to 5 years^12^, ^13^, ^14^ and in the treatment of PsA for up to 3 years.^15^, ^16^, ^17^, ^18^, ^19^ In Japanese patients, ixekizumab was efficacious and well tolerated in patients with moderate‐to‐severe psoriasis vulgaris (including PsA), as well as in patients with the GPP and EP subtypes.^20^, ^21^, ^22^

Based on clinical trial data, ixekizumab was approved in Japan in May 2016 for the treatment of psoriasis vulgaris, PsA, GPP, and EP at an initial dose of 160 mg subcutaneously followed by 80 mg at 2‐week intervals for weeks 2 to 12 and 80 mg at 4‐week intervals thereafter (Q2/Q4 dosing).^23^ Subsequently, a higher dosage of ixekizumab, 80 mg every 2 weeks after week 12 (Q2/Q2 dosing), was approved in Japan in August 2018 for patients with an inadequate response at week 12.^24^, ^25^ A citrate‐free formulation of ixekizumab, designed to reduce injection site pain, became available in September 2022.

This post‐marketing, observational study was conducted according to the requirements of the Pharmaceuticals and Medical Devices Agency in Japan and aimed to address several clinical questions regarding the long‐term use of ixekizumab in real‐world clinical practice. The primary objective was to provide real‐world data on the long‐term safety (up to 3 years) of ixekizumab in patients with psoriasis vulgaris, PsA, GPP, and EP who had inadequate responses to conventional systemic therapies under routine clinical practice. The secondary objectives were to examine the effectiveness of ixekizumab and its impact on quality of life (QoL) for up to 52 weeks from treatment commencement. As the Q2/Q2 dosing regimen is only approved in Japan, we evaluated the effectiveness and safety of Q2/Q2 dosing with reference to Q2/Q4 dosing.

To the best of our knowledge, this is the first observational study to collect safety data beyond 52 weeks in Japanese patients treated with ixekizumab. The findings from this study are likely to provide key insights regarding the clinical response to long‐term ixekizumab usage, which will be of interest and utility to health‐care providers (HCPs) who treat Japanese patients with psoriasis.

## 2 METHODS

### 2.1 Study design and participants

This was a single‐arm, prospective, non‐interventional, observational study conducted in routine clinical practice at 163 sites in Japan from November 2016 to September 2022. During the study period, the Q2/Q2 dosing regimen was approved in Japan (August 2018) for patients who had an inadequate response to ixekizumab at week 12.^24^ As the current study commenced before the release of the citrate‐free formulation, which reduces injection site pain, patients in the current analysis were not administered the citrate‐free formulation of ixekizumab.

Patients were recruited to the study via consecutive registration. Eligible patients were those diagnosed with psoriasis vulgaris, PsA, GPP, and/or EP who had inadequate responses to conventional systemic therapies and were treated with ixekizumab for the first time. Data were collected from routine clinical practice via a case report form (CRF). In total, safety and effectiveness were assessed for 3 years and 1 year respectively, commencing at the start of ixekizumab treatment. The observation period was 52 weeks. If treatment was discontinued within 52 weeks, the observation period ended 30 days after the last dose. Safety and effectiveness data were collected at baseline (week 0 before treatment commencement) and at weeks 4, 12, 24, and 52 of ixekizumab treatment (the observation period), except in the case of QoL data, which were collected at baseline and at weeks 12, 24, and 52.

The study protocol adhered to applicable local and country‐specific laws and regulations pertaining to protection of patient privacy and safety and was reviewed by the Pharmaceuticals and Medical Devices Agency. In accordance with these laws and regulations, this study did not require written informed consent from enrolled patients, and ethics approval was waived. An exception was the QoL data, where the collection and assessment of the Dermatology Life Quality Index (DLQI) scores was undertaken only for patients who provided written consent.

### 2.2 Data collection and assessments

Data collected included baseline patient demographics, clinical characteristics, and the use of ixekizumab, including dose, frequency, treatment adherence, and the reasons for change of dose and/or study discontinuation. Safety was assessed via the occurrence of adverse events (AEs) during the observation period and serious infections and malignancies that occurred during the safety follow‐up period (regardless of whether ixekizumab treatment was discontinued or not). Safety data related to serious infections and malignancies were collected for a further 2 years (safety follow‐up period). A Japan risk management plan was developed based on clinical trial data for ixekizumab, where several AEs were identified as being potentially high risk in Japanese patients and were, therefore, monitored closely throughout the study. These included serious infection, serious hypersensitivity reaction, decrease in neutrophil count, inflammatory bowel disease, malignancy, and interstitial lung disease. Measures of effectiveness included the Psoriasis Area and Severity Index (PASI), which assesses the extent of body surface involvement and the severity of psoriatic lesions on a scale of 0 (no psoriasis) to 72 (the most severe disease possible)^26^; the percentage of body BSA affected by psoriasis on a continuous scale from 0% (no involvement) to 100% (full involvement), where 1% corresponds to the size of the patient's handprint including the palm, fingers, and thumb^27^; the Disease Activity Score in 28 joints using C‐reactive protein (DAS28‐CRP) for patients with PsA, a measure of disease activity in 28 joints that consists of a composite numerical score, with higher scores indicating higher disease activity^28^; and patient‐reported QoL assessed via the DLQI, where scores range from 0 to 30, with higher scores indicating greater QoL impairment.^29^, ^30^ Safety and effectiveness measures were assessed in the safety and effectiveness analysis sets respectively, and by disease subtype and ixekizumab Q2/Q2 and Q2/Q4 doses.

### 2.3 Statistical analysis

The sample size was set with a focus on the incidence of serious infection. In clinical trials, serious infection occurred at an approximate rate of 1.6%. Assuming that the incidence of serious infection in the general population may be twice the threshold of 1.6%, a sample size of 664 patients was required with a significance level of 2.5% (one‐sided) and a power of 80% with a binomial distribution. To ensure the accuracy of estimation, the target sample size was set as 700 patients for the safety evaluation. In consideration of dropouts, the target sample size to be collected was set as 740 patients.

The safety analysis set comprised all registered patients who received at least one dose of ixekizumab. The effectiveness analysis set was performed with a subset of the safety analysis set (depending on data availability).

Statistical analyses were based on estimation, as this study was performed under routine clinical practice without a comparator. Continuous variables were summarized with descriptive statistics, including the mean, median, and standard deviation. Categorical variables, including binary variables, were summarized using frequency, incidence proportions, point estimates, and associated 95% confidence intervals. AEs, including serious AEs (SAEs), were coded using the Japanese version of the Medical Dictionary for Regulatory Activities, version 25.1. Missing values were not imputed, but the last observations were carried forward for the last visit data of the effectiveness endpoint. Statistical testing was performed as deemed appropriate. Multiplicity adjustments were not performed. Analyses were performed using SAS version 9.2 or above (SAS Institute Inc.).

## 3 RESULTS

### 3.1 Patient disposition and baseline characteristics

**FIGURE 1:**
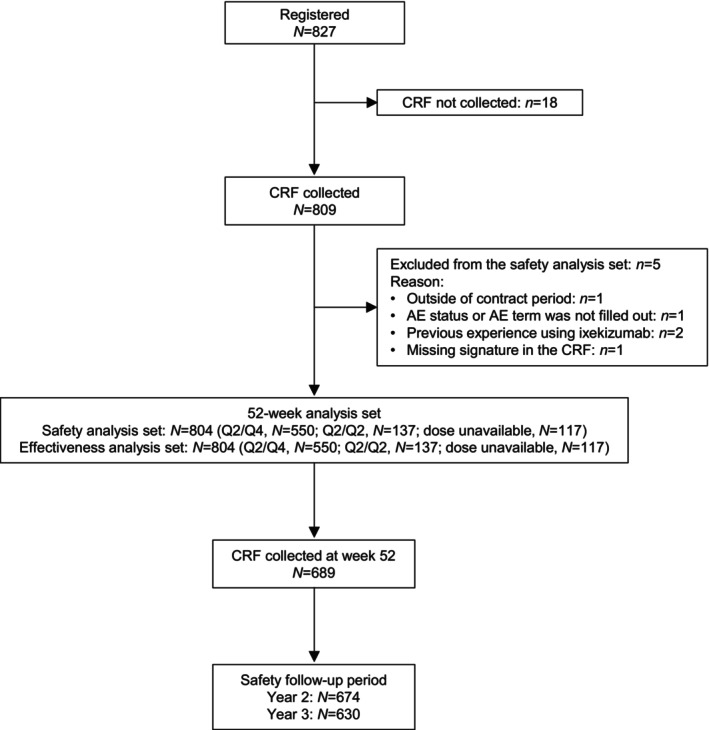
Study flowchart. AE, adverse event; CRF, case report form; Q2/Q2, an initial ixekizumab dose of 160 mg, followed by 80 mg every 2 weeks from week 12; Q2/Q4, an initial ixekizumab dose of 160 mg, followed by 80 mg every 2 weeks from week 2 to 12 and 80 mg every 4 weeks thereafter.

Of the 827 patients who were registered in the study, a CRF was not collected for 18 patients, and five patients were excluded due to violation of the contract period (*n* = 1), AE status/AE term not filled out (*n* = 1), previous experience using ixekizumab (*n* = 2), and a missing signature in the CRF (*n* = 1). Overall, 804 patients were included in the safety and effectiveness analysis sets (Figure 1).

The baseline demographics and clinical characteristics of included patients are outlined in Table 1. In total, 67.9% of patients were male, the median age was 54 years, and the median duration of psoriasis was 11.8 years. Most patients had psoriasis vulgaris (*n* = 586 [72.9%]), followed by PsA (*n* = 30 [37.7%]), GPP (*n* = 63 [7.8%]), and EP (*n* = 30 [3.7%]). The proportion of patients with two disease types is outlined in Table S1. Briefly, 149 patients (25.4%) had both psoriasis vulgaris and PsA, seven (1.2%) had both psoriasis vulgaris and GPP, and seven (1.2%) had both psoriasis vulgaris and EP. In the patients’ medical histories, hepatitis B virus was the most frequently reported previous medical condition (4.7% [*n* = 38]). Hypertension (17.5% [*n* = 141]) was the most commonly reported comorbidity among patients with psoriasis vulgaris. Most patients received prior medication for psoriasis (*n* = 675 [84.0%]), including 386 patients (48.0%) receiving biologics. Secukinumab (*n* = 120 [14.9%]) and adalimumab (*n* = 101 [12.6%]) were the most commonly used biologics prior to ixekizumab treatment (Table 1).

**TABLE 1:** Baseline demographics and clinical characteristics.

Characteristic	Total^a^ (*N* = 804)	Psoriasis vulgaris (*n* = 586)	Psoriatic arthritis (*n* = 303)	Pustular psoriasis (*n* = 63)	Erythrodermic psoriasis (*n* = 30)
Sex, *n* (%)					
Male	546 (67.9)	420 (71.7)	183 (60.4)	30 (47.6)	24 (80.0)
Female	257 (32.0)	165 (28.2)	120 (39.6)	33 (52.4)	6 (20.0)
Not described	1 (0.1)	1 (0.2)	0 (0.0)	0 (0.0)	0 (0.0)
Age, years, median (range)	54 (13–90)	53 (13–90)	52 (13–90)	56 (16–90)	55 (16–84)
Weight, kg, *n*	268	193	97	20	10
Mean (SD)	71.8 (18.2)	73.6 (17.6)	71.0 (19.7)	58.7 (15.5)	65.6 (11.6)
BMI, kg/m^2^, *n*	252	178	94	20	10
Mean (SD)	26.1 (5.8)	26.6 (5.6)	26.0 (6.3)	22.8 (5.0)	23.9 (4.0)
Psoriasis duration, years, *n*	684	503	259	53	25
Median (range)	11.8 (0.2–55.8)	12.2 (0.2–55.8)	11.0 (0.3–51.0)	10.3 (0.4–42.2)	14.0 (2.2–47.7)
Medical history, *n* (%)^b^	158 (19.7)	108 (18.4)	68 (22.4)	12 (19.1)	6 (20.0)
Hepatitis B virus	38 (4.7)	25 (4.3)	13 (4.3)	6 (9.5)	1 (3.3)
Latent tuberculosis	7 (0.9)	6 (1.0)	4 (1.3)	0 (0.0)	0 (0.0)
Drug eruption	7 (0.9)	2 (0.3)	3 (1.0)	2 (3.2)	1 (3.3)
Comorbidities, *n* (%)^c^	421 (52.4)	302 (51.5)	174 (57.4)	36 (57.1)	21 (70.0)
Hypertension	141 (17.5)	108 (18.4)	58 (19.1)	8 (12.7)	8 (26.7)
Diabetes mellitus	55 (6.8)	46 (7.9)	18 (5.9)	3 (4.8)	2 (6.7)
Hyperuricemia	54 (6.7)	42 (7.2)	19 (6.3)	3 (4.8)	4 (13.3)
Hyperlipidemia	53 (6.6)	43 (7.3)	12 (4.0)	5 (7.9)	2 (6.7)
Dyslipidemia	31 (3.9)	20 (3.4)	13 (4.3)	2 (3.2)	0 (0.0)
Prior nonpharmacological therapy for psoriasis, *n* (%)	74 (9.2)	58 (9.9)	23 (7.6)	7 (11.1)	4 (13.3)
Prior medication for psoriasis, *n* (%)	675 (84.0)	497 (84.8)	250 (82.5)	49 (77.8)	22 (73.3)
Biologic drug use, *n* (%)^d^	386 (48.0)	271 (46.3)	170 (56.1)	30 (47.6)	15 (50.0)
Secukinumab	120 (14.9)	89 (15.2)	45 (14.9)	13 (20.6)	6 (20.0)
Adalimumab	101 (12.6)	62 (10.6)	69 (22.8)	2 (3.2)	4 (13.3)
Ustekinumab	80 (10.0)	68 (11.6)	15 (5.0)	2 (3.2)	5 (16.7)
Infliximab	59 (7.3)	38 (6.5)	32 (10.6)	8 (12.7)	2 (6.7)
Brodalumab	63 (7.8)	40 (6.8)	30 (9.9)	5 (7.9)	2 (6.7)
Guselkumab	14 (1.7)	8 (1.4)	13 (4.3)	2 (3.2)	0 (0.0)
PASI score, *n*	508	387	177	26	27
Mean (SD)	11.7 (10.0)	12.0 (9.6)	9.0 (9.4)	10.9 (10.3)	21.9 (14.1)
Range	0.0–69.2	0.0–69.2	0.0–49.5	0.0–37.6	0.4–56.8
BSA, *n*	293	215	109	26	12
Mean (SD)	19.8 (21.2)	20.0 (20.0)	12.7 (17.0)	21.5 (22.8)	57.4 (28.7)

Abbreviations: BMI, body mass index; BSA, body surface area; PASI, Psoriasis Area and Severity Index; SD, standard deviation.

^a^ Patients may have more than one disease subtype.

^b^ Only the most common indications are reported.

^c^ Comorbidities reported in 3% or more of patients in the overall population.

^d^ Psoriatic medication/therapy other than ixekizumab. Presence or absence of medication used within 1 year before the start of ixekizumab treatment and discontinued before the start of ixekizumab treatment.

### 3.2 Use of ixekizumab

**FIGURE 2:**
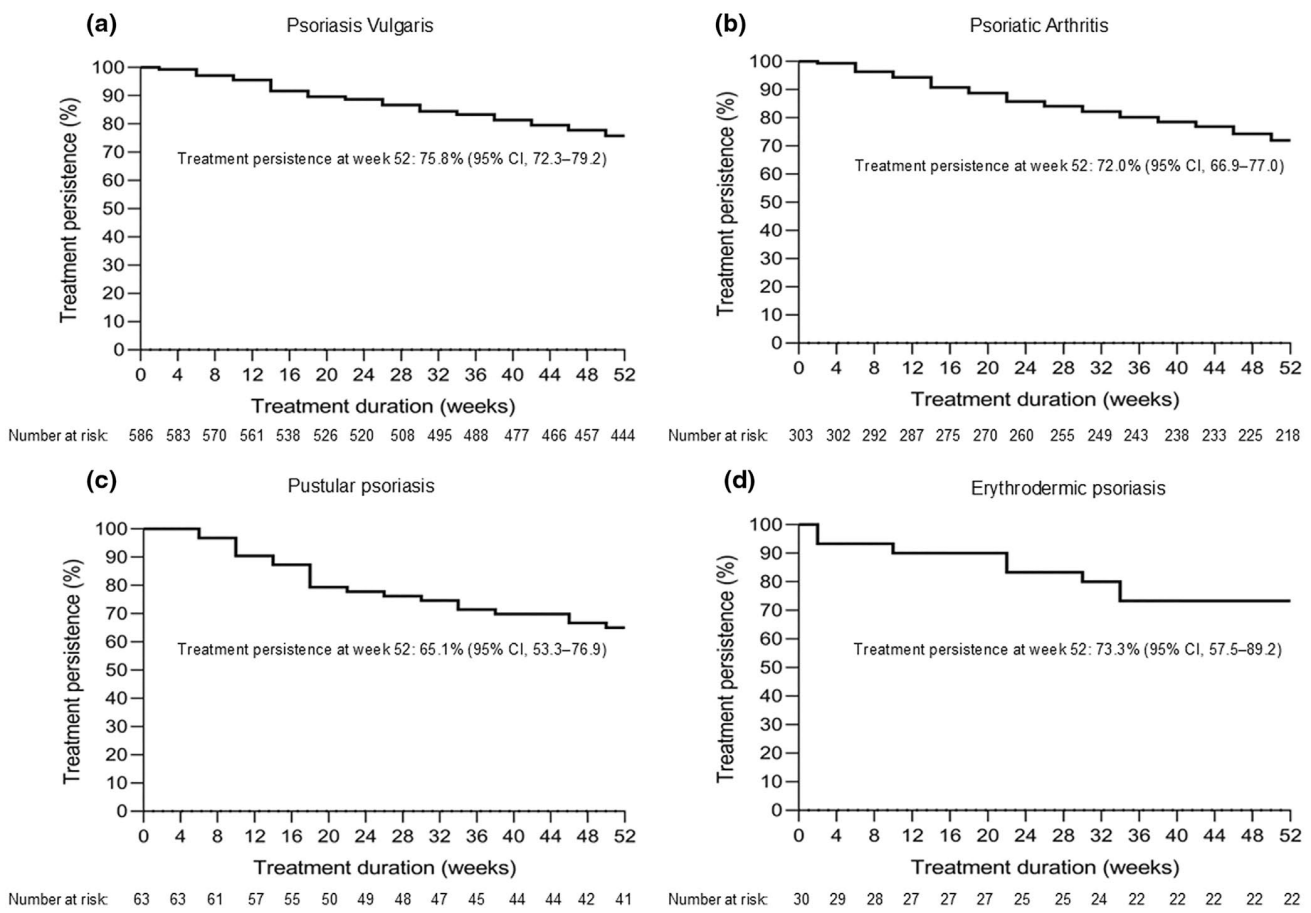
Treatment persistence of ixekizumab treatment in Japanese patients up to week 52 by disease type: (a) psoriasis vulgaris, (b) psoriatic arthritis, (c) pustular psoriasis, and (d) erythrodermic psoriasis. CI, confidence interval.

The most common device used by patients to administer ixekizumab was an autoinjector (*n* = 642 [79.9%]) followed by a prefilled syringe (*n* = 134 [16.7%]). Additionally, 28 patients (3.5%) used both devices. Overall, 137 patients (17.0%) continued with every 2‐week dosing (Q2/Q2) and 550 (68.4%) changed to every 4‐week dosing (Q2/Q4) beyond 12 weeks after the start of ixekizumab treatment. Patients who discontinued treatment before week 12 or received only one dose after week 12 (*n* = 117 [14.6%]) were excluded from the safety analysis set by ixekizumab dosing intervals (Q2/Q2 and Q2/Q4). At week 52, treatment persistence across all dosing regimens was 75.8% (*n* = 444) for patients with psoriasis vulgaris, 72.0% (*n* = 218) for patients with PsA, 65.1% (*n* = 41) for patients with GPP, and 73.3% (*n* = 22) for patients with EP (Figure 2). The most common reasons for discontinuation of ixekizumab treatment were ineffectiveness of treatment (13.9% [*n* = 112]), which predominantly occurred between ≥24 and <52 weeks, and AEs (6.5% [*n* = 52]), which mostly occurred between ≥12 and < 24 weeks (Table 2).

**TABLE 2:** Reasons for study discontinuation.

Reason for discontinuation	Discontinuation period (weeks)						Total, n (%)
	<4 4 (0.5)	≥4 to <8 22 (2.7)	≥8 to <12 14 (1.7)	≥12 to <24 65 (8.1)	≥24 to <52 103 (12.8)	≥52 46 (5.7)	254 (31.6%)
Insufficient effectiveness	1	9	6	27	52	17	112 (13.9)
Adverse event	0	6	5	16	13	12	52 (6.5)
Lost to follow‐up	2	2	1	9	13	2	29 (3.6)
Symptom improved	0	0	1	4	8	5	18 (2.2)
Death	0	0	0	0	1	0	1 (0.1)
Other	1	4	1	6	12	7	31 (3.9)

*Note*: The total number of patients in the safety population (*N* = 804) was used as the denominator for all percentages.

Baseline demographics and clinical characteristics by ixekizumab dose are outlined in Table S2. In general, baseline demographics and clinical characteristics were well balanced between patients who received the ixekizumab Q2/Q2 and Q2/Q4 doses and with the overall population. However, patients with PsA comprised a higher proportion of the Q2/Q2 dose group (56.9% [*n* = 78]) compared with the Q2/Q4 dose group (32.9% [*n* = 181]). For the remaining disease types, 62.8% (*n* = 86) and 77.5% (*n* = 426) of patients with psoriasis vulgaris received the Q2/Q2 and Q2/Q4 doses of ixekizumab respectively, while 5.8% (*n* = 8) and 7.6% (*n* = 42) of patients with GPP and 3.7% (*n* = 5) and 3.3% (*n* = 18) of patients with EP received the Q2/Q2 and Q2/Q4 doses, respectively.

### 3.3 Safety

During the 52‐week observation period, 203 patients (25.3%) experienced AEs of which 36 (4.5%) were SAEs (Table 3). Injection site reactions were experienced by 27 patients (3.4%). The incidence was 0.6% (*n* = 5) for both interstitial lung disease and serious allergy/hypersensitivity. Seven patients (0.9%) had AEs related to malignancies. Among the patients with benign, malignant, and unspecified neoplasms, gastric cancer stage I occurred in two patients, and there was one case each of rectal cancer, hepatocellular carcinoma, squamous cell carcinoma of the skin, thyroid neoplasm, and laryngeal squamous cell carcinoma. A total of 22 patients (2.7%) had AEs related to fungal infection disorders, which were considered related to ixekizumab treatment for 19 patients (2.4%). Fungal infections that occurred in two or more patients included oral candidiasis (*n* = 7 [0.9%]), tinea pedis (*n* = 7 [0.9%]), body tinea (*n* = 3 [0.4%]), dermatophytosis of the nail (*n* = 2 [0.3%]), and esophageal candidiasis (*n* = 2 [0.3%]). All cases of fungal infection were non‐serious and were resolved or resolving, except for the one case of tinea infection that occurred in one patient and in which the outcome was unknown. Alcoholic psychosis was reported by one patient (0.1%). There were no reported cases of other psychiatric disorders, including depression or suicidal ideation.

**TABLE 3:** Summary of AEs by disease type and ixekizumab dose up to week 52 of treatment.

	Overall (*N* = 804)	Disease type				Ixekizumab dose	
		Psoriasis vulgaris (*n* = 586)	Psoriatic arthritis (*n* = 303)	Pustular psoriasis (*n* = 63)	Erythrodermic psoriasis (*n* = 30)	Q2/Q2 (*n*= 137)	Q2/Q4 (*n* = 550)
Number of patients with any AE, *n* (%)	203 (25.3)	144 (24.6)	69 (22.8)	22 (34.9)	10 (33.3)	21 (15.3)	141 (25.6)
Number of patients with any SAE, *n* (%)	36 (4.5)	17 (2.9)	10 (3.3)	11 (17.5)	1 (3.3)	2 (1.5)	32 (5.8)
ADRs, *n* (%)	135 (16.8)	98 (16.7)	51 (16.8)	15 (23.8)	6 (20.0)	—	—
Serious ADRs, *n* (%)	14 (1.8)	6 (1.0)	2 (0.7)	7 (11.1)	1 (3.3)	—	—
AEs leading to discontinuation, *n* (%)	52 (6.5)	30 (5.1)	21 (6.9)	7 (11.1)	2 (6.7)	3 (2.2)	17 (3.1)
Death, *n* (%)^a^	1 (0.1)	1 (0.2)	0 (0.0)	0 (0.0)	0 (0.0)	0 (0.0)	1 (0.2)
AEs, *n* (%)							
Injection site reaction^b^	27 (3.4)	20 (3.4)	13 (4.3)	1 (1.6)	0 (0.0)	4 (2.9)	14 (2.6)
Fungal infection^c^	22 (2.7)	20 (3.4)	8 (2.6)	2 (3.2)	1 (3.3)	5 (3.7)	17 (3.1)
Oral candidiasis	7 (0.9)	7 (1.2)	1 (0.3)	0 (0.0)	0 (0.0)	1 (0.7)	6 (1.1)
Tinea pedis	7 (0.9)	7 (1.2)	3 (1.0)	0 (0.0)	1 (3.3)	2 (1.5)	5 (0.9)
Body tinea	3 (0.4)	2 (0.3)	1 (0.3)	1 (1.6)	0 (0.0)	0 (0.0)	3 (0.6)
Dermatophytosis of nail	2 (0.3)	2 (0.3)	1 (0.3)	1 (1.6)	0 (0.0)	1 (0.7)	1 (0.2)
Esophageal candidiasis	2 (0.3)	2 (0.3)	1 (0.3)	0 (0.0)	0 (0.0)	0 (0.0)	2 (0.4)
Malignant tumors	7 (0.9)	5 (0.9)	1 (0.3)	1 (1.6)	1 (3.3)	1 (0.7)	6 (1.1)
Major adverse cardiovascular events	6 (0.8)	4 (0.7)	2 (0.7)	0 (0.0)	0 (0.0)	1 (0.7)	4 (0.7)
Serious allergies and hypersensitivity	5 (0.6)	2 (0.3)	0 (0.0)	3 (4.8)	0 (0.0)	0 (0.0)	4 (0.7)
Interstitial lung disease	5 (0.6)	1 (0.2)	2 (0.7)	3 (4.8)	0 (0.0)	0 (0.0)	4 (0.7)
Inflammatory bowel disease	0 (0.0)	0 (0.0)	0 (0.0)	0 (0.0)	0 (0.0)	0 (0.0)	0 (0.0)
Serious AEs, *n* (%)							
Infections and infestations	13 (1.6)	4 (0.7)	4 (1.3)	7 (11.1)	0 (0.0)	0 (0.0)	13 (2.4)
Cellulitis	5 (0.6)	2 (0.3)	1 (0.3)	2 (3.2)	—	—	5 (0.9)
Pneumonia bacterial	3 (0.4)	2 (0.3)	1 (0.3)	1 (1.6)	—	—	3 (0.6)
Pneumonia	2 (0.3)	0 (0.0)	0 (0.0)	2 (3.2)	—	—	2 (0.4)
Sepsis	2 (0.3)	0 (0.0)	1 (0.3)	2 (3.2)	—	—	2 (0.4)
Pulmonary tuberculosis	1 (0.1)	1 (0.2)	0 (0.0)	0 (0.0)	—	—	1 (0.2)
COVID‐19	1 (0.1)	0 (0.0)	1 (0.3)	0 (0.0)	—	—	1 (0.2)
Benign, malignant, and unspecified neoplasms	6 (0.8)	4 (0.7)	0 (0.0)	1 (1.6)	1 (3.3)	—	6 (1.1)
Gastric cancer stage I	2 (0.3)	2 (0.3)	—	0 (0.0)	0 (0.0)	—	2 (0.4)
Laryngeal squamous cell carcinoma	1 (0.1)	0 (0.0)	—	0 (0.0)	1 (3.3)	—	1 (0.2)
Squamous cell carcinoma of skin	1 (0.1)	1 (0.2)	—	0 (0.0)	0 (0.0)	—	1 (0.2)
Hepatocellular carcinoma	1 (0.1)	1 (0.2)	—	0 (0.0)	0 (0.0)	—	1 (0.2)
Rectal cancer	1 (0.1)	0 (0.0)	—	1 (1.6)	0 (0.0)	—	1 (0.2)
Thyroid neoplasm	1 (0.1)	1 (0.2)	1 (0.3)	0 (0.0)	0 (0.0)	—	—
Respiratory, thoracic, and mediastinal disorders	4 (0.5)	0 (0.0)	1 (0.3)	3 (4.8)	0 (0.0)	1 (0.7)	3 (0.6)
Interstitial lung disease	2 (0.3)	—	0 (0.0)	2 (3.2)	—	—	2 (0.4)
Sleep apnoea syndrome	1 (0.1)	—	1 (0.3)	0 (0.0)	—	1 (0.7)	—
Hypersensitivity pneumonitis	1 (0.1)	—	0 (0.0)	1 (1.6)	—	—	1 (0.2)

*Note*: Data are shown for the safety analysis set. Adverse events (AEs), including serious AEs (SAEs), were tabulated using the Japanese version of the Medical Dictionary for Regulatory Activities version 25.1. AEs are shown by specific terms derived by grouping preferred terms; serious AEs are shown by system organ class and preferred term. Adverse drug reactions (ADRs) were judged by the treating physician.

Abbreviations: COVID‐19, coronavirus disease 2019; Q2/Q2, an initial ixekizumab dose of 160 mg, followed by 80 mg every 2 weeks from week 12; Q2/Q4, an initial ixekizumab dose of 160 mg, followed by 80 mg every 2 weeks from weeks 2 to 12 and 80 mg every 4 weeks thereafter.

^a^ The patient death was due to a cardiovascular event.

^b^ Included injection site pruritis, injection site erythema, injection site swelling, and injection site pain.

^c^ Fungal infections occurring in two or more patients overall are reported.

The most frequently reported SAEs were cellulitis (0.6% [*n* = 5]) and bacterial pneumonia (0.4% [*n* = 3]), followed by pneumonia, sepsis, gastric cancer stage I, and interstitial lung disease (0.3% [*n* = 2 each]). Cellulitis was considered unrelated to ixekizumab treatment in 4/5 patients reporting cellulitis, while all events of bacterial pneumonia and interstitial lung disease were considered related to ixekizumab treatment. All events were resolved or resolving at week 52. Pulmonary tuberculosis occurred in one patient with psoriasis vulgaris and was considered related to ixekizumab treatment.

Subgroup analyses by patient characteristics including sex, age, body mass index (BMI), and disease duration revealed no clinically meaningful differences in AEs compared with the total population (Table S3). Similarly, there were no substantial differences in the incidence proportion of AEs or SAEs between patients who received or did not receive prior biologic treatment (Table S4). AEs were reported by 102 patients (26.4%) taking prior biologic medication compared with 101 (24.2%) who did not take prior biologic medication, and the incidence of SAEs (prior biologic medication vs no prior biologic medication) was 6.5% (*n* = 25) versus 2.6% (*n* = 11) (Table S4). There were three adolescent patients with no reported adverse drug reactions.

The long‐term safety overview from the 2‐year and 3‐year safety follow‐up period is outlined in Table 4. The incidence proportion of serious infections and infestations was 0.0% and 0.8% (*n* = 5) during years 2 and 3, respectively. Before prescribing ixekizumab, it is recommended that screening tests be performed for certain infections. Over the course of 3 years, there were no reported serious cases of *Aspergillus*, *Cryptococcus*, or *Pneumocystis* pneumonia, and no exacerbation or new cases of hepatitis C virus. Regarding tuberculosis and hepatitis B virus by week 52, there was one new case of pulmonary tuberculosis and one case of reactivation of hepatitis B virus (one hepatitis B virus carrier had elevated hepatitis B virus DNA, which was not serious). From year 2 to year 3 there were no reports of new cases or reactivation of either tuberculosis or hepatitis B virus. The incidence of benign, malignant, and unspecified neoplasms was 0.9% (*n* = 6) at 2 years and 0.6% (*n* = 4) at 3 years. There were two cases of malignant lung neoplasm and one case of breast cancer in year 2. In year 3, there were four instances of metastases reported in three patients. The remaining diagnosed malignancies occurred in one patient each.

**TABLE 4:** Incidence of serious infections and malignancies during the safety follow‐up period.

	Year 2 (*n* = 674)	Year 3 (*n* = 630)
Infections and infestations	0 (0.0)	5 (0.8)
Cellulitis	—	1 (0.2)
Pneumonia aspiration	—	1 (0.2)
Sepsis	—	1 (0.2)
Pneumonia bacterial	—	1 (0.2)
COVID‐19	—	1 (0.2)
Benign, malignant, and unspecified neoplasms	6 (0.9)	4 (0.6)
Breast cancer recurrent	1 (0.2)	0 (0.0)
Prostate cancer	1 (0.2)	0 (0.0)
Lung adenocarcinoma stage I	1 (0.2)	0 (0.0)
Lung adenocarcinoma stage IV	0 (0.0)	1 (0.2)
Lung neoplasm malignant	2 (0.3)	0 (0.0)
Small cell lung cancer extensive stage	0 (0.0)	1 (0.2)
Hepatocellular carcinoma	0 (0.0)	1 (0.2)
Metastases to bone^a^	0 (0.0)	1 (0.2)
Metastases to kidney^a^	0 (0.0)	1 (0.2)
Metastases to liver^a^	0 (0.0)	1 (0.2)
Metastases to lymph nodes^a^	0 (0.0)	1 (0.2)
Squamous cell carcinoma of skin	1 (0.2)	0 (0.0)
Rectosigmoid cancer metastatic	0 (0.0)	1 (0.2)

*Note*: Data are presented as *n* (%) for the safety analysis set. Adverse events (AEs), including serious AEs, were tabulated by system organ class and preferred term using the Japanese version of the Medical Dictionary for Regulatory Activities version 25.1.

Abbreviations: COVID‐19, coronavirus disease 2019.

^a^ Metastases occurred in three patients overall, with one patient having metastases to the bone and kidney with the primary diagnosis being lung adenocarcinoma stage IV.

In terms of safety by ixekizumab dose, patients receiving the Q2/Q2 dose had a lower incidence of AEs (15.3% [*n* = 21]) compared with those receiving the Q2/Q4 dose (25.6% [*n* = 141]) (Table 3). Similarly, patients receiving the Q2/Q2 dose had a lower incidence of SAEs (1.5% [*n* = 2]) compared with those receiving the Q2/Q4 dose (5.8% [*n* = 32]). More patients from the Q2/Q4 dosing regimen reported serious infections compared with those receiving the Q2/Q2 dose (2.4% vs 0.0%), and the rate of injection site reactions was comparable between patients receiving ixekizumab Q2/Q4 versus Q2/Q2 doses (2.6% vs 2.9%).

### 3.4 Effectiveness

**FIGURE 3:**
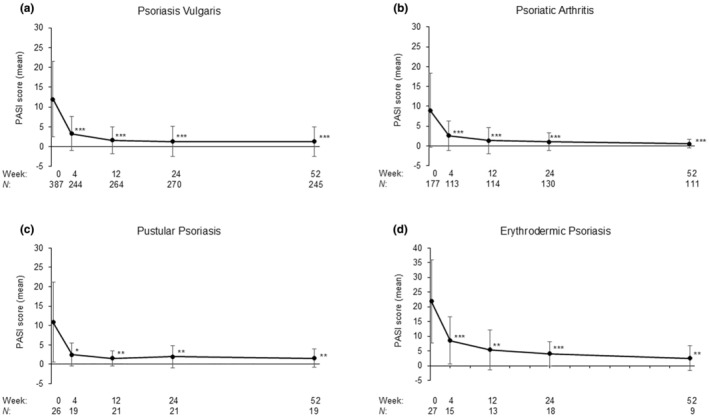
Effectiveness of ixekizumab treatment by disease type. Time course of Psoriasis Area and Severity Index (PASI) scores from baseline to week 52 by disease type: (a) psoriasis vulgaris, (b) psoriatic arthritis, (c) pustular psoriasis, and (d) erythrodermic psoriasis. Missing values were not imputed, but the last observations were carried forward for the last visit data of the effectiveness endpoint. *p < 0.05; **p < 0.01; ***p < 0.001 (*t* test).

**FIGURE 4:**
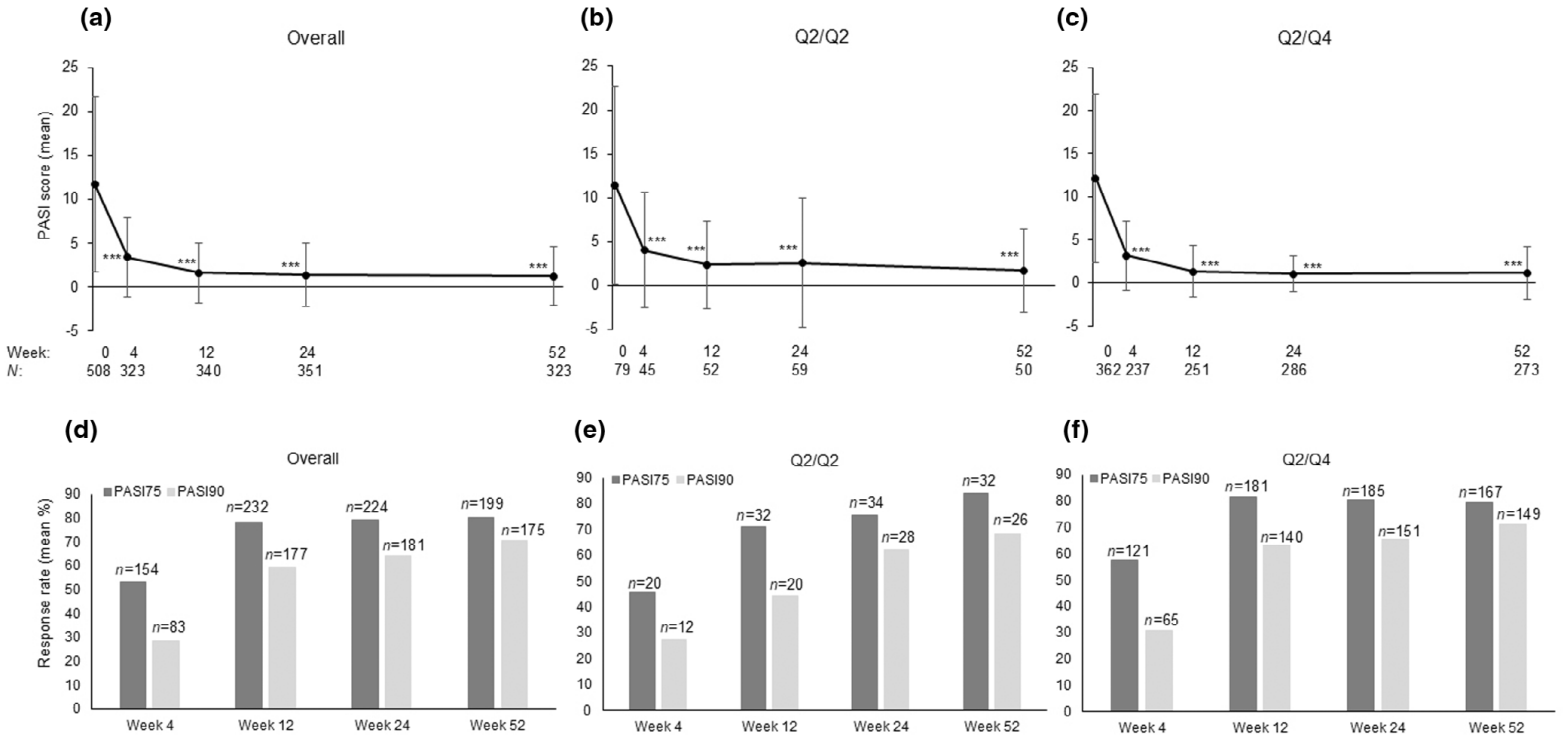
Effectiveness of ixekizumab treatment by dose. Time course of Psoriasis Area and Severity Index (PASI) scores from baseline to week 52 in the (a) overall population and by ixekizumab, (b) Q2/Q2, and (c) Q2/Q4 dose. The percentage of patients achieving PASI 75 and PASI 90 by ixekizumab dose from baseline to week 52 in the (d) overall population and by (e) ixekizumab Q2/Q2 dose, and (f) Q2/Q4 dose. Data are shown as mean (standard deviation) in panels a–c and as mean percent in panels d–f. Missing values were not imputed, but the last observations were carried forward for the last visit data of the effectiveness endpoint. Q2/Q2, an initial ixekizumab dose of 160 mg, followed by 80 mg every 2 weeks from week 12; Q2/Q4, an initial ixekizumab dose of 160 mg, followed by 80 mg every 2 weeks from weeks 2 to 12 and 80 mg every 4 weeks thereafter.

The proportions of patients who achieved PASI 50, PASI 75, PASI 90, and PASI 100 at week 52 in the overall population were 90.7%, 80.2%, 70.6%, and 56.9%, respectively. Changes in absolute PASI, PASI 75, and PASI 90 scores from week 1 to week 52 are shown in Figures 3 and 4. Significant reductions in the absolute PASI score from baseline to week 52 were observed for all psoriasis subtypes (*p* < 0.05, *p* < 0.01, or *p* < 0.001) (Figure 3). Significant reductions were observed in the absolute PASI score from baseline to week 52 in the overall population (*n* = 323) and the Q2/Q2 (*n* = 50) and Q2/Q4 (*n* = 273) populations (*p* < 0.001) (Figure 4a–c). The proportions of patients who achieved PASI 75 and PASI 90 at week 52 respectively, were 80.2% (*n* = 199) and 70.6% (*n* = 175) in the overall population, 84.2% (*n* = 32) and 68.4% (*n* = 26) in the Q2/Q2 population, and 79.5% (*n* = 167) and 71.0% (*n* = 149) in the Q2/Q4 population, respectively (Figure 4d–f). PASI 100 was achieved by 52.6% (*n* = 20) and 57.6% (*n* = 121) in the Q2/Q2 and Q2/Q4 populations, respectively. The subgroup analysis showed no substantial differences in the absolute PASI score by sex, age, body mass index (BMI), and disease duration in the overall population and by ixekizumab Q2/Q2 and Q2/Q4 doses (Figure S1), regardless of whether patients received prior biological therapy (Figure S2).

**FIGURE 5:**
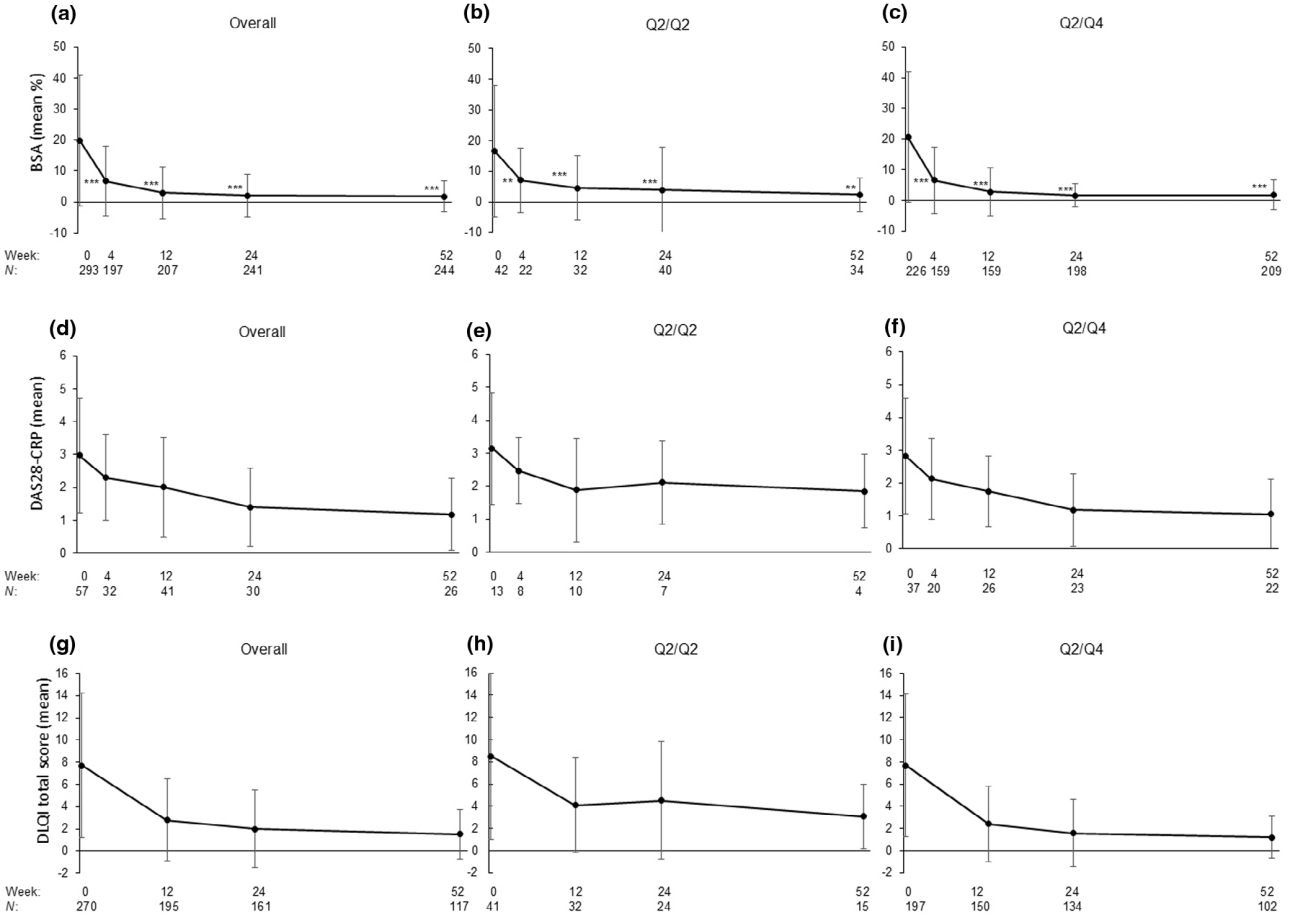
Effectiveness of ixekizumab treatment. Time course of body surface area (BSA) from baseline to week 52 in (a) the overall population and by ixekizumab, (b) Q2/Q2, and (c) Q2/Q4 dose. Disease Activity Score in 28 joints using C‐reactive protein (DAS28‐CRP) by ixekizumab dose in patients with psoriatic arthritis in (d) the overall population and by ixekizumab doses in (e) Q2/Q2, and (f) Q2/Q4 dose. Patient‐reported quality of life assessed via the Dermatology Life Quality Index (DLQI) (g) in the overall population and by (h) Q2/Q2 and (i) Q2/Q4 ixekizumab dose. Data are shown as mean (standard deviation). Q2/Q2, an initial ixekizumab dose of 160 mg, followed by 80 mg every 2 weeks from week 12; Q2/Q4, an initial ixekizumab dose of 160 mg, followed by 80 mg every 2 weeks from week 2 to 12 and 80 mg every 4 weeks thereafter. **p < 0.01; ***p < 0.001 (*t* test).

Significant reductions in absolute BSA were observed from baseline to week 52 in the overall population (*n* = 244) and by ixekizumab Q2/Q2 (*n* = 34) and Q2/Q4 (*n* = 209) doses (*p* < 0.01 or *p* < 0.001) (Figure 5a–c). Numerical decreases in DAS28‐CRP were observed in patients with PsA, although this did not reach statistical significance, potentially due to the limited number of patients (Figure 5d–f). DLQI total scores also numerically decreased from baseline to week 52 for the overall population and by ixekizumab dose (Figure 5g–i).

## 4 DISCUSSION

Real‐world evidence has become increasingly important for health‐care decision making,^31^ with real‐world data complementing clinical trial findings and providing additional data outside the constraints of clinical trial guidelines. The current analysis of post‐marketing data was conducted to provide insight regarding the safety and effectiveness of ixekizumab in Japanese patients with psoriasis vulgaris, PsA, GPP, and EP who had inadequate responses to conventional therapies under routine clinical practice. This analysis included data relating to the long‐term incidence of serious infections and malignancies of ixekizumab treatment (up to 3 years). To the best of our knowledge, this is the first observational study to collect safety data beyond 52 weeks in Japanese patients treated with ixekizumab. Furthermore, data relating to the safety and effectiveness of ixekizumab Q2/Q2 versus Q2/Q4 dosing after 1 year is reported. As the ixekizumab Q2/Q2 dose is currently only available in Japan, the safety and effectiveness findings in this population are of interest to HCPs and patients. Of note, Japanese patients with PsA in the current analysis were more likely to use the Q2/Q2 dose. It is also worth noting that treatment persistence was high (65.1%–75.8%) in the real‐world setting across all ixekizumab dosing regimens and psoriasis subtypes.

There were no new safety concerns identified in this post‐marketing study, and the observed safety profile in Japanese patients was largely consistent with the known safety profile of ixekizumab, with some differences observed in incidence.^12^, ^13^, ^14^, ^15^, ^16^, ^17^, ^18^, ^19^ The overall incidence proportion of AEs after 1 year of ixekizumab treatment was 25.3%, lower than the findings reported in the UNCOVER‐J clinical trial, where 85.9% of patients with psoriasis vulgaris and 87.5% with EP reported at least one AE.^21^ However, the incidence proportion of SAEs in the current study (2.9%) was comparable to that reported in the UNCOVER‐J trial (3.8%) for patients with psoriasis vulgaris.^21^ In the current study, a higher proportion of SAEs, including serious infections, were observed in patients with GPP in comparison to the other disease subtypes. Of note, all cases recovered or were recovering. GPP is a rare and serious condition, and evidence regarding the safety and effectiveness of ixekizumab in these patients is limited. Although most fungal infections, including oral candidiasis, were reported to be related to ixekizumab treatment, the overall incidence remained low, and all were non‐serious. Before prescribing ixekizumab, it is recommended that screening tests be performed for certain infections (e.g., fungal infection, tuberculosis). We did not observe an increase in these infections over the 3‐year study period.

The recommended dosing of ixekizumab (Q2/Q4) in the label is highly efficacious with an acceptable safety profile in most patients with moderate‐to‐severe psoriasis vulgaris.^13^ Among patients who achieved a static Physician Global Assessment score of 0 or 1 after 12 weeks of ixekizumab treatment in the phase 3 UNCOVER‐3 trial (one of the primary endpoints), some did not maintain efficacy with subsequent Q2/Q4 dosing.^13^ The benefits of continued Q2/Q2 dosing after 12 weeks of ixekizumab treatment in patients with moderate‐to‐severe psoriasis vulgaris were demonstrated in the IXORA‐P trial, where patients treated continuously with ixekizumab Q2/Q2 versus Q2/Q4 dosing had higher efficacy, but comparable safety profiles, at 52 weeks.^32^ In Japan, the ixekizumab Q2/Q2 dose was approved in August 2018 for patients with an inadequate response at week 12 of treatment,^24^, ^25^ but approval was delayed in Japan by approximately 2 years during the study period of this analysis (which may have affected the differences observed in AEs due to the longer observation period of the Q2/Q4 dosing regimen). Taken together with the clinical trial findings, it is expected that the Q2/Q2 dosing regimen will be administered to fewer patients and, in general, will be given to those with a more severe disease. These factors should be considered in the interpretation of the safety findings by dosage of ixekizumab. Overall, there was a higher incidence of AEs among patients in the ixekizumab Q2/Q4 dose group (25.6%) compared with the Q2/Q2 group (15.3%). Serious infections occurred in 2.4% of patients receiving the Q2/Q4 dose (*n* = 550), with no reported cases among patients receiving the Q2/Q2 dose (*n* = 137). Of note, slightly more older patients (aged ≥65 years) received Q2/Q4 (26.0%) dosing compared with Q2/Q2 (21.9%), potentially due to older patients being more susceptible to AEs and, hence, prescribed the longer interval dosing regimen (Q2/Q4). Furthermore, elderly patients may not be comfortable with self‐injection, and Q2/Q4 dosing is less time intensive than Q2/Q2 dosing when patients visit the hospital or other HCPs for treatment administration.

Meta‐analyses and large prospective global studies have identified an increased risk of malignancies in patients with psoriasis, particularly keratinocyte cancer, lymphomas, lung cancer, and bladder cancer.^33^, ^34^ Data from 17 controlled clinical trials of ixekizumab in adult patients with moderate‐to‐severe psoriasis vulgaris showed that the rate of malignancy was 2.0%, the most common being basal cell carcinoma.^35^ There are limited data examining the relationship between psoriasis and the risk of malignancies in Japanese patients. In a retrospective analysis of 360 Japanese patients with psoriasis, the incidence rate of malignancy was 14.4%, with the most common being colorectal cancer (20.9%) followed by skin cancer (16.4%), gastric cancer (10.4%), and lung cancer (10.4%).^36^ Although the rate of malignancy was higher in Japanese patients with psoriasis vulgaris than in the general population, the difference was not statistically significant.^36^ In the current study, 0.9% (*n* = 7) of patients developed a malignancy up to week 52 of ixekizumab treatment, and the incidence of malignancies remained low in the 2‐year (0.9% [*n* = 6]) and 3‐year (0.6% [*n* = 4]) safety follow‐up periods. More malignancies were noted in Japanese patients who received prior biologic therapy compared to those with no prior biologic therapy (1.6% vs 0.2%); however, this should be further investigated in larger cohort studies due to the limited number of cases.

Significant reductions in both PASI score and BSA percentage were observed after 52 weeks of treatment in all psoriasis subtypes and by ixekizumab Q2/Q2 and Q2/Q4 doses. Although the achievement of PASI 75 and PASI 90 was similar by ixekizumab dose, the PASI 75 response rate was slightly higher at week 52 among patients who received the ixekizumab Q2/Q2 dose. This is an encouraging finding given that the Q2/Q2 dose was administered to patients who had an inadequate response after week 12 of treatment. Although the effectiveness of ixekizumab treatment was demonstrated at week 12, continuation of the Q2/Q2 dose beyond week 12 most likely occurred in patients with PsA or may have included patients with nail or scalp involvement, which can be challenging areas to treat. Subgroup analyses in the overall population and by ixekizumab dose did not demonstrate any substantial differences in the absolute PASI score between males and females or by age, BMI, and disease duration. However, low numbers of patients in some categories should be noted. In terms of QoL, DLQI total scores numerically decreased after 52 weeks of treatment in the overall population and by ixekizumab Q2/Q2 and Q2/Q4 doses, indicating improvements in patient‐reported outcomes.

To date, there are limited real‐world studies examining the safety and effectiveness of biologics for patients with moderate‐to‐severe psoriasis vulgaris. The Psoriasis Study of Health Outcomes (PSoHO) was a global, 3‐year, observational cohort study in adults with moderate‐to‐severe psoriasis vulgaris that compared the effectiveness of anti‐IL‐17A biologics with other approved biologics.^37^, ^38^ In the PSoHO, 64.3% and 55.6% of ixekizumab‐treated patients achieved PASI 90 at 6 and 12 months, respectively.^38^ In the current study, the proportions of patients who achieved PASI 90 at week 52 were 70.6% in the overall population, 68.4% in the Q2/Q2 population, and 71.0% in the Q2/Q4 population. Differences in patient numbers and characteristics between PSoHO and the current study should be considered, particularly in regard to sex and BMI. Overall, the effectiveness of ixekizumab in real‐world settings in Japan appears to be broadly consistent with the findings in previous global real‐world studies.^37^, ^39^ Although it is not appropriate to directly compare data from post‐marketing studies with that of randomized controlled trials, findings from the current study are generally consistent with what is known from clinical trials.^12^, ^13^, ^14^, ^15^, ^16^, ^17^, ^18^, ^19^

There were three adolescent patients enrolled in the current study with no adverse drug reactions reported. There are currently few clinical studies examining pediatric psoriasis, and thus treatment options are limited and often used off‐label.^40^ Indeed, the wider literature indicates that psoriasis is not well recognized or treated among pediatric patients.^41^ A phase 3 study of ixekizumab in participants aged 6 to <18 years with moderate‐to‐severe plaque psoriasis demonstrated statistically significant improvements in skin involvement, itch, and health‐related QoL, with a safety profile comparable to that seen in adults.^42^ To the best of our knowledge, no study focusing on the safety and efficacy of ixekizumab in Japanese pediatric patients has been conducted.

This study has several limitations characteristic of post‐marketing surveillance studies, including the lack of a control group and low patient numbers in some subgroups. In terms of AEs, it is possible that these may be underestimated in the real‐world setting. It is also possible that the effectiveness observed at week 52 may be overestimated as patients who discontinued treatment due to insufficient effectiveness and those without available data were not included in the 52‐week effectiveness analyses. Furthermore, there is the possibility of selection bias as patient selection for participation relied on the decision of HCPs, which may impact the generalization of the results. Moreover, the decision to prescribe the Q2/Q2 or Q2/Q4 dose of ixekizumab relied on discussions between HCPs and patients and the consideration of each patient's medical circumstances, a process that may also introduce selection bias. The results of the subgroup analyses should be interpreted with caution because of the limited number of patients in some subgroups. Further studies are needed to confirm the specific period during which treatment discontinuation is likely to occur due to treatment ineffectiveness or AEs. Despite these limitations, we present real‐world data regarding the safety and effectiveness of ixekizumab in Japanese patients, with the sample size exceeding initial expectations. A future focus of post‐marketing research should include patients who received the citrate‐free formulation of ixekizumab.

In conclusion, this study provides real‐world evidence regarding the safety and effectiveness of ixekizumab in Japanese patients with psoriasis. Overall, the findings from this post‐marketing surveillance study are similar to those reported in global and Japanese clinical trials. Furthermore, similar effectiveness was observed between ixekizumab Q2/Q2 and Q2/Q4 doses, with no substantial differences in safety profiles identified. These findings add to the limited data from clinical practice and are likely to be informative for HCPs in Japan and worldwide.

## CONFLICT OF INTEREST STATEMENT

Hideshi Torii has received consulting fees and/or speaker's fees from AbbVie, Amgen, Bristol Myers Squibb, Eli Lilly and Company, Janssen, Kyowa Kirin, Maruho Co., Ltd., Novartis, Sun Pharma, and UCB Japan. Akimichi Morita has received research grants, consultancy fees, and/or speaker's fees from AbbVie, Amgen, Boehringer Ingelheim, Bristol Myers Squibb, Eisai, Eli Lilly Japan K.K., Janssen, Kyowa Kirin, LEO Pharma, Maruho Co., Ltd., Mitsubishi Tanabe Pharma Corporation, Nippon Kayaku, Novartis, Pfizer Japan, Sun Pharma Japan Ltd., Taiho Pharmaceutical Co., Ltd., Torii Pharmaceutical Co., Ltd., UCB Japan, and Ushio. Chie Yamamoto, Jiayi Dong, Mika Tsujimoto, Takashi Matsuo, and Hitoe Torisu‐Itakura are full‐time employees of Eli Lilly Japan K.K. Chie Yamamoto, Mika Tsujimoto, Takashi Matsuo, and Hitoe Torisu‐Itakura are stockholders of Eli Lilly and Company. Mamitaro Ohtsuki has received research grants and/or personal fees from AbbVie, Bristol Myers Squibb, Eisai, Eli Lilly and Company, Janssen, LEO Pharma K.K., Maruho Co., Mitsubishi Tanabe Pharma Corporation, Novartis, Taiho Pharmaceutical, and Torii Pharmaceutical. Hidehisa Saeki has received grants or contracts from AbbVie GK, Eisai Co., Ltd., LEO Pharma K.K., Maruho Co., Ltd., Sun Pharma Japan Ltd., Taiho Pharmaceutical Co., Ltd., and Torii Pharmaceutical Co., Ltd., and honoraria for lectures from AbbVie GK, Amgen, Bristol Myers Squibb, Eli Lilly Japan K.K., LEO Pharma K.K., Maruho Co., Ltd., Mitsubishi Tanabe Pharma Corporation, Nippon Boehringer Ingelheim, Novartis Pharma K.K., Taiho Pharmaceutical Co., Ltd., Torii Pharmaceutical Co., Ltd., and UCB Japan.

Hidehisa Saeki is an Editorial Board member of *The Journal of Dermatology* and a co‐author of this article. To minimize bias, he was excluded from all editorial decision‐making related to the acceptance of this article for publication.

## ETHICS STATEMENT

This survey was conducted in compliance with the Good Post‐marketing Study Practice (GPSP Ordinance) (Ministry of Health, Labour and Welfare Ordinance No. 171, dated December 20, 2004). The study protocol adhered to applicable local and country‐specific laws and regulations pertaining to protection of patient privacy and safety and was reviewed by the Pharmaceuticals and Medical Devices Agency. In accordance with these laws and regulations, this study did not obtain written informed consent from enrolled patients and ethics approval was waived. An exception was the quality of life data, where the collection and assessment of Dermatology Life Quality Index scores were only undertaken for patients who provided written consent.

## Supporting information


Data S1.

## Data Availability

Due to the observational nature of this research, consent to share data publicly could not be obtained from the participants of this study, therefore, supporting data are not available.
