# Supplementary material for: Safety and effectiveness of ixekizumab in Japanese patients with psoriasis vulgaris, psoriatic arthritis, generalized pustular psoriasis, and erythrodermic psoriasis: Post‐marketing surveillance
Source: J Dermatol. 2025 Mar 13;52(5):787–801. doi: 10.1111/1346-8138.17695 (PMC12056272; doi:10.1111/1346-8138.17695)
Supplement: Supplementary file 1 — Data S1. [file JDE-52-787-s001.docx]

**SUPPLEMENTARY APPENDIX**

**TABLE S1.** Proportion of patients with 2 disease types.

|  | **Psoriasis vulgaris**  **(*N*=586), *n* (%)** | **Psoriatic arthritis**  **(*N*=303), *n* (%)** | **Pustular psoriasis**  **(*N*=63), *n* (%)** | **Erythrodermic psoriasis**  **(*N*=30), *n* (%)** |
| --- | --- | --- | --- | --- |
| Psoriasis vulgaris |  | 149 (49.2) | 7 (11.1) | 7 (23.3) |
| Psoriatic arthritis | 149 (25.4) |  | 14 (22.2) | 6 (20.0) |
| Pustular psoriasis | 7 (1.2) | 14 (4.6) |  | 2 (6.7) |
| Erythrodermic psoriasis | 7 (1.2) | 6 (2.0) | 2 (3.2) |  |

**TABLE S2.** Patient demographics and clinical characteristics by ixekizumab dose.

| **Characteristic** | **Total**  **(*N*=804)** | **Q2/Q2 (*N*=137)** | **Q2/Q4**  **(*N*=550)** |
| --- | --- | --- | --- |
| Sex, *n* (%) |  |  |  |
| Male | 546 (67.9) | 94 (68.6) | 386 (70.2) |
| Female | 257 (32.0) | 43 (31.4) | 164 (29.8) |
| Not described | 1 (0.1) | 0 (0.0) | 0 (0.0) |
| Age, years, median (range) | 54 (13–90) | 54 (18–84) | 53 (13–90) |
| Weight, kg, *n* | 268 | 48 | 183 |
| Mean (SD) | 71.8 (18.2) | 74.6 (20.0) | 72.1 (17.7) |
| BMI, kg/m^2^, *n* | 252 | 46 | 170 |
| Mean (SD) | 26.1 (5.8) | 27.1 (6.6) | 26.1 (5.5) |
| Psoriasis duration, years, *n* | 684 | 113 | 474 |
| Median (range) | 11.8 (0.2–55.8) | 11.1 (0.4–43.0) | 12.5 (0.2–55.8) |
| With medical history, *n* (%)^†^ | 158 (19.7) | 25 (18.3) | 113 (20.6) |
| With comorbidities, *n* (%)^‡^ | 421 (52.4) | 71 (51.8) | 292 (53.1) |
| Prior therapy for psoriasis, *n* (%) | 74 (9.2) | 14 (10.2) | 46 (8.4) |
| Prior medication for psoriasis, *n* (%) | 675 (84.0) | 116 (84.7) | 465 (84.6) |
| PASI score, *n* | 508 | 79 | 362 |
| Mean (SD) | 11.7 (10.0) | 11.4 (11.3) | 12.1 (9.8) |
| BSA, %, *n* | 293 | 42 | 226 |
| Mean (SD) | 19.8 (21.2) | 16.5 (21.5) | 20.7 (21.2) |
| Disease type, *n* (%)^§^ |  |  |  |
| Psoriasis vulgaris | 586 (72.9) | 86 (62.8) | 426 (77.5) |
| Psoriatic arthritis | 303 (37.7) | 78 (56.9) | 181 (32.9) |
| Pustular psoriasis | 63 (7.8) | 8 (5.8) | 42 (7.6) |
| Erythrodermic psoriasis | 30 (3.7) | 5 (3.7) | 18 (3.3) |

Abbreviations: BMI, body mass index; BSA, body surface area; PASI, Psoriasis Area and Severity Index; Q2/Q2, an initial ixekizumab dose of 160 mg, followed by 80 mg every 2 weeks from week 12; Q2/Q4, an initial ixekizumab dose of 160 mg, followed by 80 mg every 2 weeks from weeks 2 to 12 and 80 mg every 4 weeks thereafter; SD, standard deviation.

^†^Medical history includes Crohn’s disease, ulcerative colitis, liver disorder, and renal disorder.

^‡^Comorbidities include Crohn’s disease, ulcerative colitis, liver disorder, and renal disorder.

^§^Patients could have more than 1 disease type.

**TABLE S3.** Safety overview by patient characteristics at week 52 of ixekizumab treatment.

|  | **Number of patients in category** | **Adverse events, *n* (%)** |
| --- | --- | --- |
| All patients | 804 | 203 (25.3) |
| Sex |  |  |
| Male | 546 | 124 (22.7) |
| Female | 257 | 79 (30.7) |
| Age, years |  |  |
| <65 | 599 | 139 (23.2) |
| ≥65 | 205 | 64 (31.2) |
| BMI, kg/m^2^ |  |  |
| <18.5 | 11 | 3 (27.3) |
| ≥18.5 to <22.0 | 54 | 12 (22.2) |
| ≥22.0 to <25.0 | 61 | 18 (29.5) |
| ≥25.0 to <30.0 | 69 | 12 (17.4) |
| ≥30.0 | 57 | 12 (21.1) |
| Unknown | 552 | 146 (26.5) |
| Psoriasis duration, years |  |  |
| <1 | 37 | 11 (29.7) |
| ≥1 to <5 | 114 | 25 (21.9) |
| ≥5 to <10 | 139 | 33 (23.7) |
| ≥10 to <15 | 109 | 28 (25.7) |
| ≥15 to <20 | 94 | 25 (26.6) |
| ≥20 to <25 | 68 | 21 (30.9) |
| ≥25 | 123 | 34 (27.6) |
| Unknown | 120 | 26 (21.7) |

*Note:* Data are presented for the safety analysis set.

Abbreviation: BMI, body mass index.

**TABLE S4.** Summary of AEs by prior biologic therapy use at week 52 of ixekizumab treatment.

|  | **Overall**  **(*N*=804), *n* (%)** | **Prior biologic therapy use**  **(*N*=386), *n* (%)** | **No prior biologic therapy use**  **(*N*=418), *n* (%)** |
| --- | --- | --- | --- |
| All AEs | 203 (25.3) | 102 (26.4) | 101 (24.2) |
| Serious AEs | 36 (4.5) | 25 (6.5) | 11 (2.6) |
| AEs leading to discontinuation | 52 (6.5) | 26 (6.7) | 26 (6.2) |
| Death | 1 (0.1) | 1 (0.3) | 0 (0.0) |
| Serious infection | 13 (1.6) | 9 (2.3) | 4 (1.0) |
| Injection site reaction^†^ | 27 (3.4) | 13 (3.4) | 14 (3.4) |
| Serious allergy and hypersensitivity | 5 (0.6) | 3 (0.8) | 2 (0.5) |
| Interstitial lung disease | 5 (0.6) | 3 (0.8) | 2 (0.5) |
| Inflammatory bowel disease | 0 (0.0) | 0 (0.0) | 0 (0.0) |
| Malignant tumors | 7 (0.9) | 6 (1.6) | 1 (0.2) |
| Major adverse cardiovascular events | 6 (0.8) | 3 (0.8) | 3 (0.7) |

*Note:* Data are presented for the safety analysis set. AEs, including serious AEs, were tabulated using the Japanese version of the Medical Dictionary for Regulatory Activities version 25.1.

Abbreviation: AE, adverse event.

^†^Included injection site pruritis, injection site erythema, injection site swelling, and injection site pain.

**
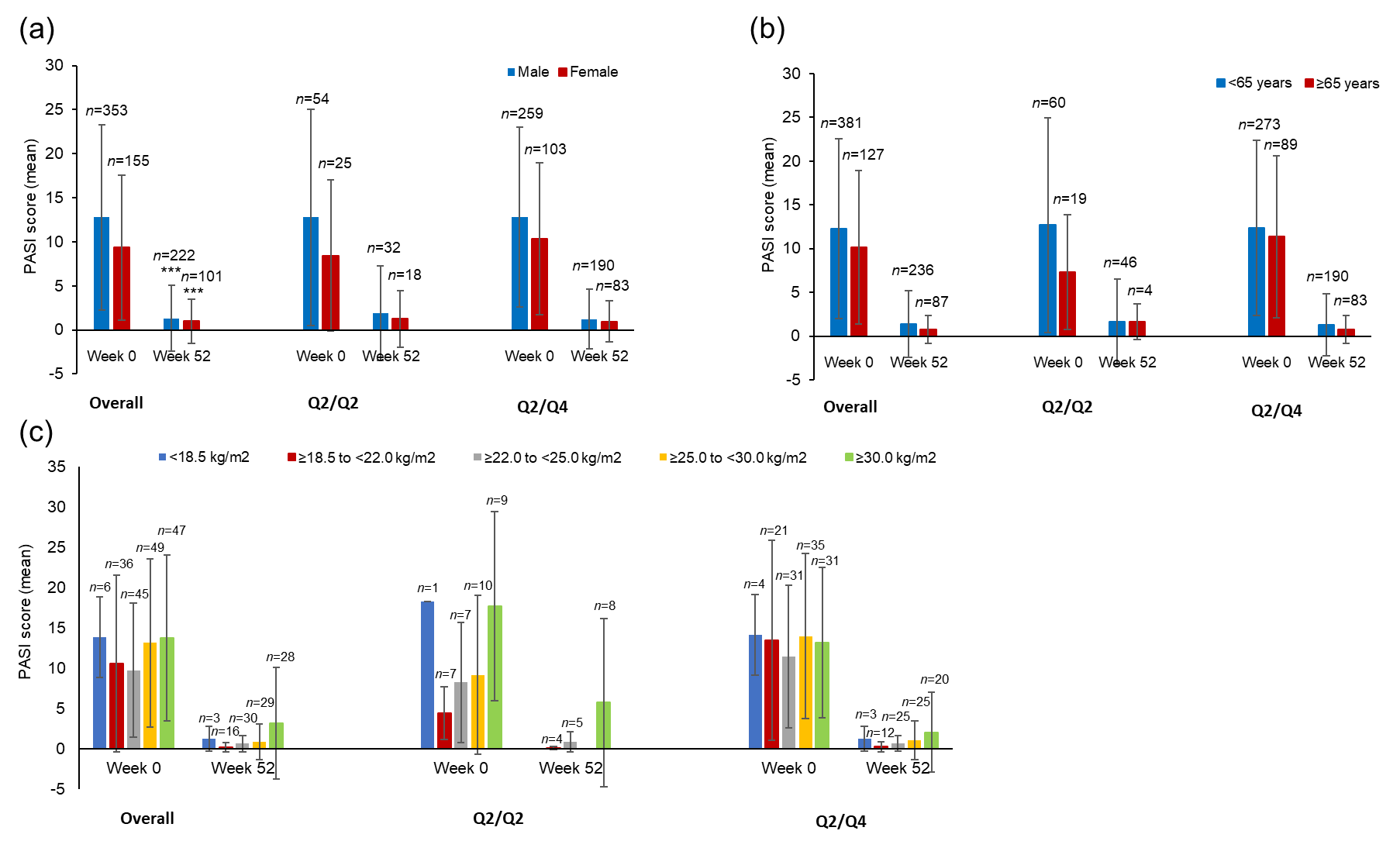
**

**
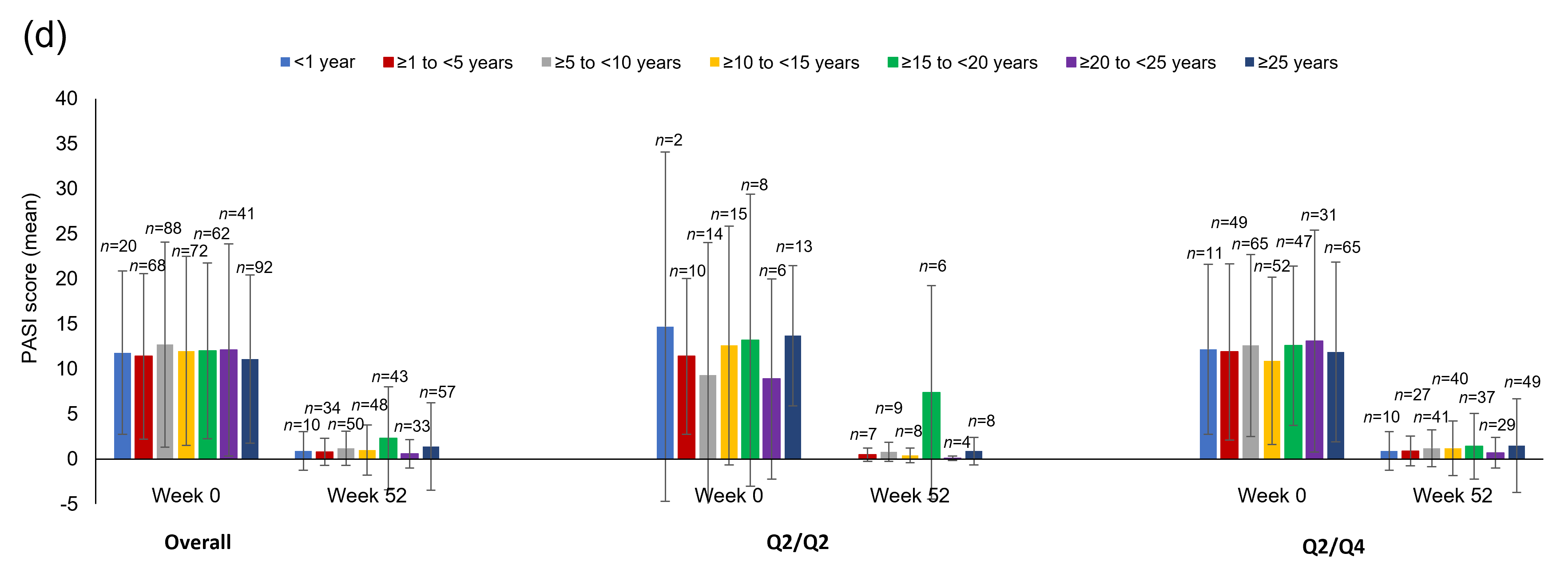
**

**FIGURE S1.** Effectiveness of ixekizumab by patient baseline characteristics. PASI scores at weeks 0 and 52 by patient baseline characteristics and ixekizumab dose by (a) sex; (b) age (<65 and ≥65 years); (c) BMI category (<18.5, ≥18.5–<22.0, ≥22.0–<25.0, ≥25.0–<30.0, and ≥30 kg/m^2^); and (d) disease duration category (<1, ≥1–<5, ≥5–<10, ≥10–<15, ≥15–<20, ≥20–<25, and ≥25 years). Data are shown as mean (standard deviation). ****p*≤0.001 (*t* test). BMI, body mass index; *n*, number of patients in the specified category; PASI, Psoriasis Area and Severity Index; Q2/Q2, an initial ixekizumab dose of 160 mg, followed by 80 mg every 2 weeks from week 12; Q2/Q4, an initial ixekizumab dose of 160 mg, followed by 80 mg every 2 weeks from weeks 2 to 12 and 80 mg every 4 weeks thereafter.

**
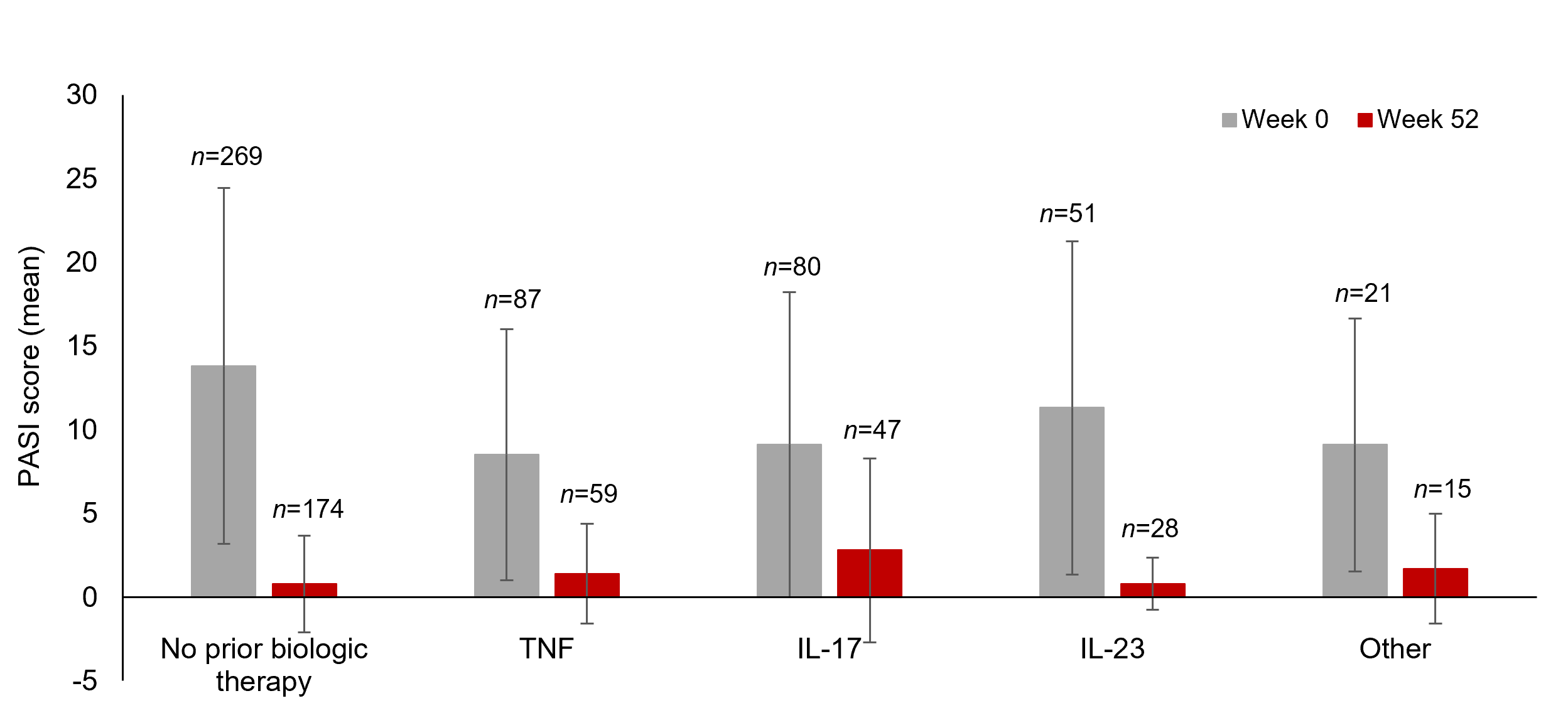
**

**FIGURE S2.** Effectiveness of ixekizumab by prior biological therapy. Absolute PASI score in patients who did not receive prior biological therapy and in patients who received TNF inhibitors, IL-17 inhibitors, IL-23 inhibitors, or other prior biological therapy at weeks 0 and 52. Data are shown as mean (standard deviation). IL, interleukin; PASI, Psoriasis Activity and Severity Index; TNF, tumor necrosis factor.
